# What is important to adults after lower limb reconstruction surgery: a conceptual framework

**DOI:** 10.1007/s11136-022-03340-7

**Published:** 2023-01-07

**Authors:** Heather Leggett, Arabella Scantlebury, Catherine Hewitt, Hemant Sharma, Catriona McDaid, The PROLLIT Study Collaborators, Joy Adamson, Kim Cocks, Paul Harwood, David Ferguson, Reggie Hamdy, Nando Ferreira

**Affiliations:** 1 York Trials Unit, The University of York, York, UK; 2 Hull University Teaching Hospitals, Hull, UK

**Keywords:** Lower limb reconstruction, Quality of life, Conceptual framework, Qualitative, Patient-reported outcome measures

## Abstract

**Purpose:**

Patient-reported outcome measures (PROMs) are used to understand the impact of lower limb reconstruction on patient’s Health-Related Quality of Life (HRQL). Existing measures have not involved this group of patients and their experiences during development. This study aimed to develop a conceptual framework to reflect what is important to patients requiring, undergoing or after undergoing reconstructive surgery.

**Methods:**

Our population of interest was people requiring, undergoing or after undergoing reconstructive surgery due to trauma, malunion, nonunion, infection or congenital issues treated by internal or external fixation. We undertook semi-structured interviews with patients and orthopaedic healthcare professionals (surgeons, methodologists and patient contributors) in England.

**Results:**

Thirty-two patients and 22 orthopaedic healthcare professionals (surgeons, methodologists and patient contributors) were interviewed between November 2020 and June 2021. Eight domains from a previously developed preliminary conceptual framework were used as a framework around which to code the interviews using thematic analysis. Six domains important to patients (from the perspective of patients and orthopaedic healthcare professionals) were included in the final conceptual framework: *pain, perception-of-self, work and finances, daily lifestyle and functioning, emotional well-being, and support*. These findings, plus meetings with our advisory panel led to the refinement of the conceptual framework.

**Conclusion:**

The first five domains relate to important outcomes for patients; they are all inter-related and their importance to patients changed throughout recovery. The final domain—*support* (from work, the hospital, physiotherapists and family/friends)—was vital to patients and lessened the negative impact of the other domains on their HRQL. These new data strengthen our original findings and our understanding of the domains we identified in the QES. The next step in this research is to ascertain whether current PROMs used with this group of patients adequately capture these areas of importance.

**Supplementary Information:**

The online version contains supplementary material available at 10.1007/s11136-022-03340-7.

## Introduction

The PROLLIT (Patient-Reported Outcome Measure for Lower Limb Reconstruction) study aims to ascertain whether current Patient-Reported Outcome Measures (PROMS) used with Lower Limb Reconstruction (LLR) patients are fit for purpose and adequately capture outcomes that are important to patients, including the development of a new PROM if required [1]. A recent qualitative evidence synthesis (QES) of nine studies using thematic synthesis highlighted the paucity of research exploring the outcomes important to people undergoing LLR [2]; this QES has enabled the preliminary identification of domains for a conceptual framework for this group of patients.

LLR in adults encompasses a range of surgical interventions including limb lengthening and deformity correction. These interventions are used for conditions including congenital abnormalities, neoplasia (development of tumours), trauma, infection or arthritis [3]. LLR can be a prolonged treatment pathway and patients may have already had significant and multiple surgical interventions, due to trauma or may have lived with their condition for some time. After surgery, patients may experience reduced mobility and independence, increased anxiety, depression, post-traumatic stress disorder, prolonged pain and detrimental effects on their work, social life, body image and identity [4, 5]. It is important for health professionals to understand patient’s experiences of LLR, recovery and Health-Related Quality of Life (HRQL). HRQL refers to the “health aspects of quality of life, generally considered to reflect the impact of disease and treatment on disability and daily functioning; it has also been considered to reflect the impact of perceived health on an individual’s ability to live a fulfilling life" [6, p. 68]. PROMs can be used by health professionals to assess the impact on a patient’s HRQL and physical functioning as well as their experiences of the injury or condition, rehabilitation and recovery [7]. PROMs are also important for assessing the effectiveness of interventions in research studies.

Current PROMS that are potentially relevant for this patient group include anatomically specific measures to assess musculoskeletal function such as the Olerud-Molander Ankle Score (OMAS) [8], and the PROMIS Physical Function 8a Short Form for people who have experienced orthopaedic trauma to a lower extremity [9] as well as non-disease-specific tools such as the Disability Rating Index [10]. PROMs can also include generic measures which assess broader health-related quality of life such as the Sickness Impact Profile (SIP) [11], the Short-Form-36 (SF-36) [12] and the Nottingham Health Profile (NHP) [13]. None of the PROMs currently used with this population have been specifically developed with the input of adults requiring a LLR [14]. Therefore, it is uncertain whether the tools being used capture the unique experiences and recovery process of adults undergoing LLR.

The QES identified a paucity of research in general on the perspectives of people undergoing LLR and the impact of their condition and surgery on their lives. The most relevant study we identified was the conceptual framework developed by Mundy et al. [15] which included a mixed population of people undergoing lower limb reconstruction surgery or an amputation in the United States. This study has fed into our preliminary conceptual framework but it was important to proceed with our primary qualitative study as planned given the very different healthcare systems and since Mundy et al. included a relatively small number (*n* = 15) of patients undergoing reconstruction surgery. Also, they took a predominantly numerical approach to presenting the data, whereas our planned thematic approach adds to the richness of the data available from this population.

The study reported in this paper aims to explore what is important to patients during and after LLR to develop and refine a conceptual framework, building on a QES to further develop a preliminary conceptual framework identifying what is important to patients [2].

## Methods

### Design

The study consisted of (1) a qualitative study undertaking semi-structured interviews with patients and orthopaedic healthcare professionals in the UK, analysed thematically, supported by an existing preliminary framework; (2) stakeholder meetings with an advisory panel, including orthopaedic surgeons, methodologists and patient and public involvement (PPI) members and (3) conceptual framework development and refinement using the findings from 1 and 2. There are no formal guidelines for conceptual framework development; however, we followed the process used in previous research [16, 17] by combining top down (QES) and bottom up data (qualitative interviews), followed by sense-checking these findings with key stakeholders. Ethical approval was given from South Central—Berkshire Research Ethics Committee (ref:20/SC/0114) and also received HRA Approval (IRAS: 269088).

### Sampling and recruitment

Patient participants were recruited from three major trauma hospitals in England. In keeping with current methodological guidance, we aimed for maximum variation in our sample, rather than saturation [18]. We used convenience sampling but aimed for maximum variation according to age, gender, reason for reconstruction, type of reconstruction and length of time since reconstruction for patients.

Patients were included if they were adults (16 +) requiring, undergoing or having undergone reconstructive surgery for a lower limb condition (leg, ankle, foot), due to a congenital or acquired condition, from trauma, infection, nonunion or malunion. Conditions could also include leg length discrepancy or bone loss, joint contracture, lower limb injuries where further limb reconstruction was required and poly-trauma patients (as long as one of the above criteria were met). Participants with an external or internal fixation were eligible.

Patient participants were identified by the clinical lead at each site. Patients were informed about the study by a clinical member of staff at the hospital and given an information sheet. Those who were interested in participating were asked to sign a consent to contact form which was passed onto the research team.

To ensure maximum variation of perspectives from healthcare professionals, we included a range of professions (frame nurse specialist, physiotherapist, orthopaedic surgeon) from different locations. Orthopaedic healthcare professionals were recruited via an email invitation from the clinical lead at each of the three NHS sites. Orthopaedic healthcare professionals were also recruited from across the UK through adverts sent out via the British Limb Reconstruction Society. All email invites contained a participant information sheet, a copy of the consent form and the researcher’s contact details; interested participants were asked to contact the research team. Consent was obtained via an online consent form before the interview.

### Data collection

Data were collected between November 2020 and July 2021. Interviews were led by a topic guide (supplementary file 1) which was used flexibly and had been informed by the QES [2], discussions within the research team and our advisory panel. Patients were asked questions which explored their thoughts, attitudes and beliefs surrounding what is important to them with regard to HRQL in relation to requiring, undergoing or after reconstructive surgery for a lower limb condition. Orthopaedic healthcare professionals were asked to discuss what they perceive to be important treatment outcomes and goals for LLR patients.

All interviews were undertaken by an experienced qualitative research fellow (HL), remotely via video conferencing or telephone; the interviewer conducted the interviews from a private room to ensure confidentiality. The interviewer had had no prior contact or relationship with the participants. Participants were allocated a participant ID which was used to identify them. Audio-recordings were deleted after transcription. Transcripts are stored on a University encrypted device and will be kept for a minimum of 5 years.

### Qualitative analysis

**Fig. 1:**
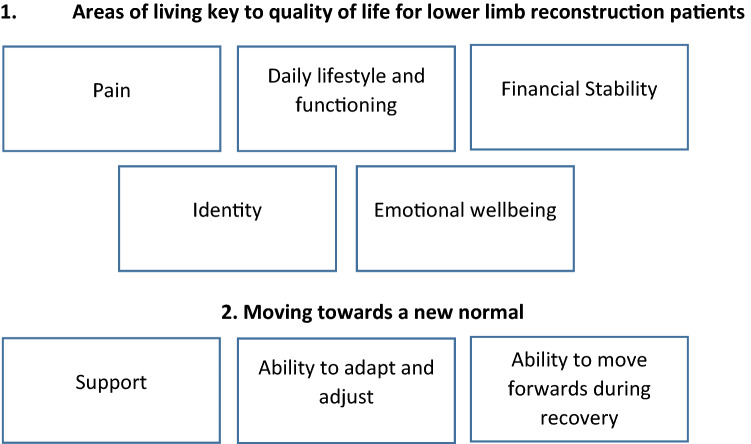
Domains identified in the QES framework

Interviews were transcribed verbatim and imported into Nvivo (V12) to aid data management. Thematic analysis [19] was undertaken and transcripts were analysed deductively using the HRQL-related domains identified in the QES framework [2]: *pain, identity, income, daily lifestyle and functioning, emotional wellbeing, support, ability to adapt and adjust* and *ability to move forwards* (Fig. 1). Within each of the HRQL-related domains, analysis was partly inductive as new codes were identified. The transcripts were coded according to the QES domains and questions posed in the topic guide. Following this, themes and sub-themes were revisited and refined to ensure that data represented areas of importance to patients (from the perspectives of both patients and orthopaedic healthcare professionals) and could be used to inform the development of potential domains in our conceptual framework. Analysis was undertaken by one researcher (HL), and code and theme development were regularly discussed with another researcher (AS) throughout the analysis. Researcher bias was minimised through iterative coding and self-reflections of the researcher (using memos, mind mapping). During analysis, two of the initial domains, *ability to adapt and adjust* and *ability to move forwards*, were removed from the conceptual framework as they focussed on elements that helped/hindered patients move forwards after LLR (e.g. coping, motivation and acceptance) rather than the impact of surgery on HRQL.

### Stakeholder meetings

After analysis, we invited members of our clinical and methodological advisory panel and PPI group to a meeting (one with each group) to gain their thoughts and perspectives on the refined framework. These were held via Zoom meetings between January and March 2022. Whilst some aspects were discussed by all groups (e.g. the temporal aspect of each domain and the inclusion of the support domain), the focus of each meeting was tailored to gain the most from the expertise of each group and covered queries that had arisen during analysis. For example, the PPI group focussed on the terminology used, the meeting with the surgeons focussed on the population the framework represented and terminology from a clinical perspective, and the meeting with the methodologists concentrated on the design of the framework. Nine stakeholders participated: 3 public members (2 males with frames, 1 female with internal fixation), 4 orthopaedic surgeons (3 from the UK and from one North America) and 2 methodologists (one qualitative researcher, one statistician).

## Results

### Participant interviews

Thirty-two patients participated (12 female and 20 male): 29 had experienced a trauma injury and 3 had a congenital condition (Table 1). All had experienced lower limb reconstruction surgery: 26 external fixation, 4 internal fixation, 2 external and internal. Twenty-two orthopaedic healthcare professionals participated: 11 physiotherapists, 4 frame specialist nurses and 7 surgeons (Table 2). Eight healthcare professionals were from the three hospital sites. Patient interviews lasted between 30 and 90 min and healthcare professional interviews 30 and 60 min.

**Table 1:** Patient characteristics

Participant ID	Gender	Type of reconstruction	Reason for reconstruction	Length of time since reconstructive surgery
P01	M	Frame (external)	Sporting injury	2 months
P02	M	Frame (external)	Congenital	18 months
P03	F	Frame (external)	Sporting injury	6 months
P04	M	Frame (external)	Fall	1 month
P05	F	Frame (external)	Osteomyelitis and Charcot joint	8 months
P06	M	Frame (external)	Road traffic accident	6 months
P07	M	Frame (external)	Aggravated an old knee injury	24 months
P08	M	Frame (external)	Ongoing issues following a previous accident	20 months
P09	M	Frame (external)	Congenital, curved arches	9 months
P10	F	Frame (external)	Sporting injury	3 months
P11	F	Plate (internal)	Old sporting injury- nonunion	8 months
P12	M	Frame (external)	Fall	1 month
P13	M	Frame (external)	Accident- heavy load at work	Not recorded
P14	F	Frame (external)	Sporting injury	19 months
P15	M	Frame(external)	Fall	5 months
P16	M	Frame (Second frame after previous infection) (external)	Sporting injury	17 months
P17	M	Frame (external)	Sporting injury	18 months
P18	M	Frame (external)	Road traffic accident	18 months
P19	M	Frame and internal fixation (external and internal)	Heavy load incident at work	30 months
P20	M	Frame (external)	Fall	3 months
P21	M	Frame (external)	Sporting injury	18 months
P22	M	Intermedullary nails (internal)	Accident- heavy load at work	3 months
P23	F	Frame (external)	Fall	7 months
P24	F	Frame (external)	Fall	2 months
P25	M	Frame (external)	Fall	13 months
P26	F	Frame (external)	Road traffic accident	6 months
P27	F	Frame (external)	Fall	11 months
P28	F	Plates (internal)	Fall	12 months
P29	M	Frame and plates (external and internal)	Fall	4 months
P30	M	Frame (external)	Accident- heavy load at work	4 months
P31	F	Nail (internal)	Fall	5 months
P32	F	Frame (external)	Road traffic accident	31 months

**Table 2:** Healthcare professional characteristics

Participant ID	Job role
HCP01	Physiotherapist
HCP02	Consultant surgeon
HCP03	Consultant surgeon
HCP04	Physiotherapist
HCP05	Consultant surgeon
HCP06	Physiotherapist
HCP07	Physiotherapist
HCP08	Consultant surgeon
HCP09	Physiotherapist
HCP10	Consultant surgeon
HCP11	Physiotherapist
HCP12	Frame Nurse specialist
HCP13	Consultant surgeon
HCP14	Frame Nurse specialist
HCP15	Consultant surgeon
HCP16	Frame Nurse specialist
HCP17	Physiotherapist
HCP18	Physiotherapist
HCP19	Physiotherapist
HCP20	Physiotherapist
HCP21	Physiotherapist
HCP22	Frame Nurse specialist

**Fig. 2:**
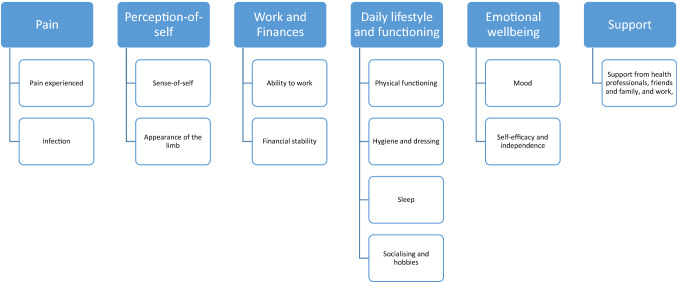
Overview of the conceptual framework

The thematic analysis led to the identification of six themes. These themes represent outcome domains within the conceptual framework which are important to patients’ HRQL after a LLR (Fig. 2) The domains identified are consistent with those in the preliminary conceptual framework developed from the QES (note that i*dentity* is now labelled *perception-of-self,* as decided in the stakeholder meetings during conceptual framework refinement). Each domain is inter-related and the importance of each domain and its interaction with other domains varied temporally over patients’ recovery journey. Table 3 provides a summary of each theme along with supporting quotes. Summary tables highlighting temporal changes, the relationship between domains and supporting quotes are provided in supplementary file 2.

**Table 3:** Summary and quotes for each domain

Domain	Summary	Quotes
Pain	All patients experienced pain at some point during their recovery. However, for most this reduced as they recovered. Pin site infections were a concern for frame patients but they found they got used to managing these and being able to recognise the onset of infection early	“Sometimes the pain went but then sometimes it could sky rocket, it could. It was ridiculous … there were days where I couldn’t literally put my foot down.” P19
		“I got an infection in my pin site, it was hell. They said actually it’s just a very sore and irritated one but they gave me the antibiotics, so thank god it wasn’t infected. But all I can say, if that wasn’t infected Christ knows what it must feel like when they are infected.” P10
Perception-of-self	Impact on sense-of-self varied between patients greatly, with some feeling as though they lost what made them ‘them’ through the loss of hobbies and work. Whereas others did not feel at all affected. Some patients were concerned about their appearance due to scars or the frame and would avoid wearing shorts due to this or avoided busy places	“I’m one of those 60 year olds that will get up and do anything scary. I mean my grandchildren say, I dare you to do that grandma, I’d do it but now I can’t… I’m just like a disabled lady walking with one leg high and one leg low.” P23
		“After having the frame off I still feel quite nervous about going outside. I’ve not been wearing shorts because I’ve got scars galore on my leg now and I think the scars caused by the frame, as I say, have set me back a little bit.” P18
Work and Finances	Impact on work depended on the patient’s job. Those who did manual labour jobs were more likely to take longer to return to work and some had to find new jobs. Those who could work from home were likely to return to work more quickly. However, some patients said they did not feel in the right mindset to work during this time even though they could have done. The loss of work and worries about financial stability increased as patients’ recovery time increased	“Work wise is a nightmare because I can’t go back to construction and I’m kind of looking at office jobs at the moment but that’s never been me, so work kind of worries me to be honest….I’ve never worked since the accident. I’m looking for work now but before I was literally on crutches for 2 years so I couldn’t get about. I couldn’t drive anywhere so I was just restricted really but now I’m quite a bit more mobile, I’m looking for work now.” P19
		“No, I’ve just put that to the back of my mind. I’m not worrying about the financial thing aspect of it all. I’ve got enough to worry about without that. So no I’m not.” P03
Daily lifestyle and functioning	Physical functioning, hygiene and dressing, socialising and hobbies, and sleep were impacted by the reconstruction and were general areas of frustration for patients. Limits to physical functioning negatively impacted other daily activities that required mobility. The impact of this usually reduced as patients recovered and became more mobile and independent	“I’m just generally worried that it’s not…. I want it to go back to how it was, and I was told when the frame was fitted that there was no reason why it won’t be as strong as it was before, and I am starting to doubt that. I’m starting to sort of think am I ever going to run? Am I ever going to play football with my daughter? Am I ever going to go on the trampoline again?” P27
		“I am absolutely exhausted. At 4 o’clock I am exhausted and when I’m sleeping, I’m out for the count.” P10
Emotional well-being	Patients were likely to experience some episodes of low mood during their recovery. This was often related to frustrations at their lack of independence, being in pain and not being able to engage in their usual pastimes. This tended to improve as recovery progressed and patients regained their independence and self-efficacy. For those with extra complications or who took longer than expected to recover this was impacted for longer	“Being bleak about it, honestly soul destroying. It really kind of took me….I’d say I’m still recovering from it because it was hard having a cage on and looking after my little boy, expecting to try and walk around with him and I couldn’t and there were days when I didn’t want to get out of bed and there were days where I didn’t want to be here anymore.” P18
		“Your independence you miss it. Miss to be able to walk and get something.”P29
Support	Support was important to all patients and could be from health professionals (both in the acute period and longer term), from family and friends and from work. Feeling informed and in control of their recovery pathway was also important to patients. Feeling informed and supported mediated the impact the other domains had on patients’ quality of life	“I don’t want to sound big headed but I’ve only been able to do that [rehabilitated well] because of the team what looked after me originally. They said you could ring up at any time and it was immediate and when I went to see [name] on three occasions he just said, carry on, you know. So I knew I was doing right.” P16
		“She’s [patients wife] my private nurse really. She’s very observant, you know, she sees that….because when I had the cage on it’s a 360 degree system and she was very knowledgeable, became very knowledgeable about what was improved and what was going backwards. She did all my dressings every day.” P09

### The domains of the conceptual framework

#### PAIN

##### Pain experienced

Pain could negatively affect emotional well-being relating to feeling depressed, unhappy, vulnerable and being dependent on others. Some found that they had not realised how much their pain had impacted their emotional well-being until they were in less pain and found they felt happier, more independent and more confident in their abilities.

##### Infection

Pin site hygiene and managing infections were very important for frame patients. Infection influenced patient outcomes through the pain and worries they caused. Patients found infections greatly added to the pain they were already experiencing and influenced their ability to be mobile. Some also limited their activities to avoid exposing themselves to potential infection or irritating the area. However, patients became more confident in managing pin sites as their recovery progressed.

#### Perception-of-self

##### Sense-of-self

Often, how participants viewed themselves was impacted during their recovery and this was especially the case for those who had experienced a life-changing traumatic injury. This usually resulted from not being able to engage in their usual pastimes, or because they were more cautious now. Sense-of-self could change over time, with patients not realising the impact of the reconstruction until they reflected on it when they had recovered. Others believed that although they experienced lifestyle changes and found daily activities harder, these did not constitute a change in their overall identity. Some participants experienced a positive impact on their sense-of-self as it caused them to have a slower pace of life and appreciate the small things that were important to them. Patients also reported making a conscious effort not to become only identifiable by their injury/condition, they recognised that it was part of them but did not want it to become them.

##### Appearance of the limb

Whilst not universal, some expressed concerns around the appearance of the lower limb after reconstruction. This influenced what clothing they felt comfortable wearing and the activities they felt confident engaging in. For some this was embarrassment about their appearance but for others it was more about being “*fed-up” [P21]* of being stared at or having to answer questions about their limb. Younger patients with a frame were particularly self-conscious when out in public.

The orthopaedic healthcare professionals described how it was common for the lower limb to look different after reconstruction, but they believed that most patients were not too concerned with long-term scaring. For those who had undergone skin grafts this could result in them feeling as though the grafted skin was alien to them. Countering this feeling however was a sense of gratefulness that their limb had been salvageable.

#### Work and Finances

##### Ability to work

Ability to work changed throughout recovery and was also dependent on occupation, security of employment, type of injury and nature of the surgery. Patients found that their concerns around returning to work and future job prospects increased as their recovery time increased. Inability to work could negatively impact emotional well-being, their perception-of-self and was a source of frustration for patients, especially those who were particularly concerned about income, self-employed or enjoyed their job. Those with more physical jobs were likely to be off work for longer, with some anticipating that a job change would be required. Those who could work from home or were office based were more likely to return to work sooner. However, it was also common for patients to delay their return or be concerned about returning to work; patients described a *“brain fog” [P10]* which prevented them from focussing or concentrating properly.

Positively, returning to work aided participants in reclaiming their perception-of-self and their independence; however, it could also have negative outcomes for patients. Some patients felt as though their return had been too soon, they felt *“burnt out” [P11]* and found the pressure of working whilst recovering overwhelming. Others felt the physical effects of returning to work and experienced increased pain from being active all day or sitting at a desk. However, when they were ready, returning to work or embarking on a new career was a significant outcome, marking an important stage in recovery and a return to normality.

##### Financial stability

Those who were on paid sick leave, were retired, had been able to claim on insurance or received a compensation pay-out were less concerned about their income. Income did become a concern if patients were self-employed, had lost their job, or were no longer on paid sick leave. Initially after surgery, patients were less worried about money and were instead trying to focus on getting better. However, as the length of time off work increased so too did financial concerns. Those with fewer savings and/or a lack of pay during this period were openly worried about money and discussed how they had begun applications for financial aid or were being helped out by family.

#### Daily lifestyle and functioning

##### Physical functioning

The impact of the LLR on patient’s mobility was greatest immediately after surgery, especially for older patients, those who experienced a more severe injury or had reduced mobility pre-reconstruction. Patients were also concerned about the future regarding their physical functionality and mobility. This was a worry for patients if it stopped them from engaging in their usual activities. Those for whom physical functioning and mobility improved during recovery noted that this helped them regain their perception-of-self, independence, return to work, socialise and participate in their hobbies. However, some patients had not regained full mobility and reported that they were unlikely to do so. Continued poor mobility and pain led to some patients expressing regret that they had not had an amputation.

##### Hygiene and dressing

Washing and dressing were key to regaining or maintaining independence. Most patients experienced difficulties maintaining hygiene and dressing early on in their recovery and had to rely on others for help. There was a learning curve for patients and as their recovery progressed they were often able to make adaptions and modifications such as using a stool to get in and out of the bath, creating a waterproof device or buying something to cover the leg completely so that it stayed dry.

Patients who underwent an external fixation found dressing particularly difficult as they struggled to get clothes to fit over their frames. Patients became quite creative with modifying clothes: many cut up trousers to get them over the frame and added wider sections of material or velcro to enable the frame to be covered.

##### Sleep

For a number of patients, especially those with a frame, quality and quantity of sleep were negatively affected during recovery. Patients found that they needed to be more well-rested otherwise this impacted their mood and overall well-being. It was a shock and an annoyance for patients that bedclothes clung to the frame and they found that they inadvertently ripped bedding during the night. It was common for patients to sleep in a separate bed to their partners for various reasons: fear they may hurt them with the frame, so that they had more room or because they were restless. Regardless of the type of reconstruction, quantity of sleep was impacted; patients found that they needed to sleep for longer than usual and that they often felt exhausted, even if they had not been physically active.

##### Socialising and hobbies

Limitations on physical functioning, confidence in going outside and mood affected patient’s ability and interest in undertaking their usual hobbies and socialising. Inability or a lack of desire to undertake usual activities could further negatively impact patient’s emotional well-being. Patients went through stages of not feeling up to socialising and also became tired at the same conversations or focus on their limb. In particular, those who said they experienced low mood, anxiety and/or struggled with coping reported withdrawing from social interactions during this time. A number of patients took solace in social activities during their recovery or had been able to return to hobbies or find new ones they enjoyed.

#### Emotional well-being

##### Mood

Many patients experienced low mood at some point during their recovery and reported feeling depressed, unhappy, sad or frustrated. This was often related to being in pain, immobile, feeling unsupported, struggling to be independent and feeling isolated. For most patients, these feelings resolved as their recovery progressed and they became more mobile and independent as their pain reduced. However, for others, these feelings did persist and were particularly exacerbated if they experienced a step backwards in their recovery due to infection or pain or were not recovering at the rate they expected to. Younger patients in particular felt as though their life was on hold whilst they were recovering as they could not live as they would usually do. Some often felt left behind as their friends and family were moving on without them.

##### Self-efficacy and independence

Many participants experienced a lack of confidence in their ability to walk either at all or long distances and had a fear of falling over. This was often more pronounced for those who lived alone as they felt more vulnerable if they were to have an accident. A lack of confidence could negatively affect participant’s perception-of-self through its impact on their engagement in daily activities and hobbies. Resultantly, feeling confident was an important outcome to patients because they were more likely to become mobile, independent and find their “*new normal” [P32]*. It was also common for patients to experience worries or anxieties during their recovery regarding their recovery progress, re-injuring themselves, leaving the house, their ability to work and worrying whether there would be any long-term consequences or complications.

Particularly at the beginning of their recovery, participants were dependent on friends and family for help with daily tasks and activities; this reliance on others could lead to feelings of guilt that they were burdening others. Participants also felt as though their life was on hold during recovery and that they had lost important time in their life. Trauma patients were more likely to feel guilty; some felt it was their fault they were in this situation. An important outcome for patients therefore was regaining their independence and becoming more confident in undertaking daily activities alone, being mobile, as well as finding their *‘new normal’.*

Being motivated and setting goals were also key to gaining independence and returning to *“some normality” [P02]*. Patients’ reported goals included returning to sport, going on holiday, returning to work, playing with children, grandchildren or pets or walking unaided again. Physiotherapists played an important role as they encouraged patients and supported them to set goals. It was important that goals were flexible and could be modified depending on the stage of the patient’s recovery—a goal set straight after surgery would be very different to a goal set when the patient was regaining their mobility.

#### Support

Participants who felt supported were often those who had more positive experiences across the domains presented in this framework. These patients were more likely to discuss better pain management, fewer work and financial worries, less impact on emotional well-being and being better able to cope with the impact of their injury on daily lifestyle and mobility. Patients who felt well supported were also more likely to feel motivated and set goals to work towards. Support came from a variety of sources and included family and friends, employers and healthcare professionals. Patients discussed immediate hospital support after the LLR, longer-term hospital support during recovery and support through physiotherapy. It was important to patients that support continued through the recovery journey, not just at the acute stage. Feeling informed and having good knowledge of what was to be expected during recovery and where to seek help from was also important to patients; these patients felt more confident in their next steps in the recovery process.                                       

### Stakeholder meetings and conceptual framework refinement

There were a number of revisions made to the framework between its development through the QES [2] and the final framework presented here. These changes arose based on our analysis, research team meetings and stakeholder meetings. The domain named *perception-of-self* was originally entitled *identity*. It became apparent during analysis and our post-analysis stakeholder meeting with the PPI group that *identity* was too strong a phrase and did not accurately represent those who did not feel as though their identity had changed or those who felt that a change in identity was too strong a description for what they experienced. During analysis and throughout discussions with each stakeholder group, the importance of the temporal aspect of each domain was highlighted. Consequently, the change in impact or importance of each domain over time has been made clearer in the final conceptual framework. Another key discussion point in all stakeholder meetings was around the inclusion of *support*. The domain of *support* was included as although it is not an ‘outcome’, feeling supported was important to all participants and appeared to act as a buffer to other domains. Although it may not be part of an eventual outcome measure, stakeholders thought it was important to capture in the framework.

## Discussion

This research explored what was important to patients undergoing a LLR in relation to their HRQL, with the aim of developing a conceptual framework to represent this. The final conceptual framework represents six domains which reflect key areas of importance for patients: *pain, perception-of-self, work and finances, daily lifestyle and functioning, emotional wellbeing, and support*. These new data strengthen our original findings and our understanding of the domains we identified in the QES.

Our findings overlap with earlier research including studies in the earlier QES [2]. One of the studies included in the QES reported a conceptual framework developed based on interviews with a mixed population of 33 participants from a single institution who had undergone LLR, amputation or both procedures following a trauma [20]. There is some overlap with our framework in the areas identified, in particular in demonstrating the importance of appearance, satisfaction, finances, physical functioning, psychological functioning, impact on social functioning and impact on work. There are also some areas unique to both frameworks, such as prothesis (reflecting the different population) and sexual functioning in Mundy et al.’s framework and the impact on perception-of-self, pain, and the importance of support in ours. Our study provides further insight into and richness of data on the experiences of patients undergoing a LLR and areas important to them during their recovery. The differences between the two studies may also reflect the different populations that they were developed with (single United States site, amputation and reconstruction patients in the Mundy study compared to three England sites, orthopaedic healthcare professionals and reconstruction patients). This is something to keep in mind when developing a PROM for patients undergoing a LRR as a ‘one size fits all’ approach may not be appropriate.

Our framework showed that support was very important to patients and impacted the influence of the other domains on patient’s outcomes. Those who felt supported (from health professionals, loved ones and work) were more likely to have a better quality of life during recovery due to the impact this had on their trust in their clinical team, their ability to cope with recovery, and fewer worries around work and income. For example, patients who felt supported by their clinical team were confident that help was on hand if they had any issues or concerns during recovery. Those who felt supported by their employer were less worried about their length of time to return to work or their financial situation. In this sense, support was a moderator for the potential negative effects that the recovery period could contribute to in the other domains. Previous research illustrates the importance of support for LLR patients [21–24]. This information on support, which captures patient experience rather than health or well-being outcome [25], has the potential to be useful to clinicians when providing care to patients. As this framework highlights key areas important to patients and key difficulties that patients may face it could facilitate important conversations between health professionals and patients around these areas. Future analysis of the dataset will further explore the role of patient experience and support on recovery and the implications for patient care.

### Strengths and limitations

A strength of our sample was that it included patients at different stages of recovery at the time of interview. This gave us insight as to how the impact of LLR on a patient’s HRQL differed and changed throughout recovery. For example, most patient’s mobility improved as their recovery progressed and they found it easier to undertake daily activities independently. It was important to capture these changes in our framework since PROMs can be used to assess patient change over time. Unsurprisingly, we found that the domains in this framework affected patients differently throughout their recovery. The conceptual framework was also strengthened by the triangulation of perspectives from both patients and healthcare professionals from across England enabling insights into patient experiences from both perspectives and allowed viewpoints to be corroborated or a different viewpoint on the patient perspective offered. Importantly, we found that patients and health professionals had very similar views on the importance of each domain.

The majority of patients in our sample had had an external frame fixation following an acute injury. This may reflect the fact that we recruited from major trauma centres and that fewer elective surgeries were being undertaken during the recruitment period due to the COVID-19 pandemic. This is mitigated to an extent as healthcare professionals were able to give their perspective on the wide range of patients they provided care to. Unfortunately, we did not collect data on the age of participants or health professionals years of experience limiting any exploration of these dimensions.

## Conclusion

This paper presents a conceptual framework which reflects HRQL outcomes important to adult patients undergoing LLR surgery. Five outcome domains were identified *(pain, perception-of-self, work and finances, daily lifestyle and functioning and emotional well-being and)* with 12 sub-domains. There was a sixth domain, *support*, which underpinned patients’ recovery. The next stage in our research is to ascertain whether the PROMs currently used with this group of patients capture these important domains.

## Supplementary Information

Below is the link to the electronic supplementary material.

Supplementary file1 (PDF 197 kb)

Supplementary file2 (PDF 214 kb)

Supplementary file3 (PDF 481 kb)

## Data Availability

The datasets generated during and/or analysed during the current study are available from the corresponding author on reasonable request.

## References

[1] Leggett H, Scantlebury A, Sharma H, Hewitt C, Harden M, McDaid C (2020). Quality of life following a lower limb reconstructive procedure: A protocol for the development of a conceptual framework. British Medical Journal Open.

[2] Leggett H, Scantlebury A, Byrne A, Harden M, Hewitt C, O'Carroll G, Sharma H, McDaid C (2021). Exploring what is important to patients with regards to quality of life after experiencing a lower limb reconstructive procedure: A qualitative evidence synthesis. Health and Quality of Life Outcomes.

[3] Burton M, Walters S, Saleh M, Brazier J (2012). An evaluation of patient-reported outcome measures in lower limb reconstruction surgery. Quality of Life Research.

[4] Castillo RC, MacKenzie EJ, Wegener ST, Bosse MJ (2006). Prevalence of chronic pain seven years following limb threatening lower extremity trauma. Pain.

[5] McCarthy ML, MacKenzie EJ, Edwin D, Bosse MJ, Castillo RC, Starr A (2003). Psychological distress associated with severe lower-limb injury. Journal of Bone and Joint Surgery. American Volume.

[6] Mayo, N. E. (2015). *Dictionary of quality of life and health outcomes measurement* (1st ed.). ISOQOL.

[7] Black N (2013). Patient reported outcome measures could help transform healthcare. BMJ: British Medical Journal.

[8] Olerud C, Molander H (1984). A scoring scale for symptom evaluation after ankle fracture. Archives of Orthopaedic and Traumatic Surgery.

[9] Rothrock NE, Kaat AJ, Vrahas MS, O'Toole RV, Buono SK, Morrison S, Gershon RC (2019). Validation of PROMIS physical function instruments in patients with an orthopaedic trauma to a lower extremity. Journal of Orthopaedic Trauma.

[10] Salen BA, Nordemar R (1994). The Disability Rating Index: An instrument for the assessment of disability in clinical settings. Journal of clinical epidemiology.

[11] Bergner M, Bobbitt RA, Pollard WE, Martin DP, Gilson BS (1976). The sickness impact profile: Validation of a health status measure. Medical care.

[12] Ware JE (2000). SF-36 health survey update. Spine.

[13] Hunt SM, McKenna SP, McEwen J, Williams J, Papp E (1981). The Nottingham Health Profile: Subjective health status and medical consultations. Social Science & Medicine. Part A.

[14] Antonios T, Barker A, Ibrahim I, Scarsbrook C, Smitham PJ, Goodier WD, Calder PR (2019). A systematic review of patient-reported outcome measures used in circular frame fixation. Strategies in Trauma and Limb Reconstruction.

[15] Mundy LR, Klassen A, Grier AJ, Gibbons C, Lane W, Carty MJ, Pusic AL, Hollenbeck ST, Gage MJ (2020). Identifying factors most important to lower extremity trauma patients: Key concepts from the development of a patient-reported outcome instrument for lower extremity trauma, the LIMB-Q. Plastic and reconstructive surgery.

[16] Gorecki C, Lamping DL, Brown JM, Madill A, Firth J, Nixon J (2010). Development of a conceptual framework of health-related quality of life in pressure ulcers: A patient-focused approach. International Journal of Nursing Studies.

[17] Gorecki C, Brown JM, Nelson EA, Briggs M, Schoonhoven L, Dealey C, Defloor T, Nixon J (2009). Impact of pressure ulcers on quality of life in older patients: A systematic review. Journal of the American Geriatrics Society.

[18] Braun V, Clarke V (2021). To saturate or not to saturate? Questioning data saturation as a useful concept for thematic analysis and sample-size rationales. Qualitative research in sport, exercise and health.

[19] Braun, V., & Clarke, V. (2021). *Thematic analysis: A practical guide*. Sage Publications Ltd.

[20] Mundy LR, Klassen A, Grier AJ, Gibbons C, Lane W, Carty MJ, Pusic AL, Hollenbeck ST, Gage MJ (2020). Identifying factors most important to lower extremity trauma patients: key concepts from the development of a patient-reported outcome instrument for lower extremity trauma. The LIMB-Q. Plastic and Reconstructive Surgery.

[21] Bernhoff K, Bjorck M, Larsson J, Jangland E (2016). Patient experiences of life years after severe civilian lower extremity trauma with vascular injury. European Journal of Vascular & Endovascular Surgery.

[22] Tutton, E., Achten, J., Lamb, S. E., Willett, K., & Costa, M. L. (2018). A qualitative study of patient experience of an open fracture of the lower limb during acute care. B*one & Joint Journal, 100-B(*4), 522–526. 10.1302/0301-620X.100B4.BJJ-2017-0891.R1 29629594

[23] Griffiths H, Jordan S (1998). Thinking of the future and walking back to normal: An exploratory study of patients' experiences during recovery from lower limb fracture. Journal of Advanced Nursing.

[24] Phelps EE, Tutton E, Griffin X, Baird J (2019). A qualitative study of patients’ experience of recovery after a distal femoral fracture. Injury.

[25] Bull C, Teede H, Watson D, Callander EJ (2022). Selecting and implementing patient-reported outcome and experience measures to assess health system performance. JAMA Health Forum.

