# Supplementary material for: What is important to adults after lower limb reconstruction surgery: a conceptual framework
Source: Qual Life Res. 2023 Jan 7;32(6):1671–82. doi: 10.1007/s11136-022-03340-7 (PMC9825128; doi:10.1007/s11136-022-03340-7)
Supplement: Supplementary file 1 — Supplementary file1 (PDF 197 kb) [file 11136_2022_3340_MOESM1_ESM.pdf]

Supplementary file 1. Topic guides

TOPIC GUIDE. Interviews with Patients

At the beginning of all interviews

- The qualitative researcher will introduce themselves to the participant as part of the research team.
- The qualitative researcher will explain the study and the purposes of the interview
- The qualitative researcher will explain that we would like to audio-record the interview and explain the processes for ensuring anonymity and confidentiality of interview data.
- The qualitative researcher will explain how interview data will be used.
- The qualitative researcher will determine if the participant would like to take part in the study and if so, will obtain verbal and written consent. If the participant would no longer like to take part they will be thanked for their time and the interview will not proceed.
- Participants will be provided with the opportunity to ask any questions.

This topic guide summarises the main areas to be explored for each interview. As with any qualitative interview, these headings are intended as a starting point to ensure the primary issues are covered, whilst allowing flexibility for new issues to emerge.

Main interview:

- Can you tell me a little bit about your injury? (when, how did it happen?)
- Can you tell me about the treatment you have received so far? Initial presentation; x-ray and 'diagnosis'; 'type of treatment received'
    - Experience of non-union, deformity or infection and associated limb reconstruction?
- Were you surprised by any aspect of your care? Have you experienced any difficulties so far?

- Tell me about the time after your treatment?
  With regards to:
    - Acute/rehabilitation?
    - Support?
    - What would have helped you?
    - Need of support today?

- How long do you think it will take you to recover? Do you think you will make a complete recovery?
- Please describe how you experience a regular day?
- Can you tell me how you experience life after your injury?
  With regards to:
    - Socially, family and friends / Interaction with family / Friends
    - Home activities
    - At work
    - Hygiene

    - Hobbies
    - Sleep
  - Intimacy
  - Well-being/how happy you feel?
  - Pain & Suffering

- Thinking about your life, well-being and functional activities, do you feel it has influenced your emotional well-being/how happy you feel?
- Thinking about your life, well-being and functional activities, are you worried that your injury will have any long term consequences/may stop you from doing anything you want to do?
- Are you concerned about doing anything in the future?
- Is there anything else you want to tell me?
- How does it feel to talk about this?

End of interview

- Thank participant and ask if they have any comments
- Explain again about how data will be used and reiterate about anonymity and confidentiality
- Provide opportunity for questions and state that the lead researcher is contactable after the interview, should questions arise.

TOPIC GUIDE. Interviews with Healthcare professionals

At the beginning of all interviews

- The qualitative researcher will introduce themselves to the participant as part of the research team.
- The qualitative researcher will explain the study and the purposes of the interview
- The qualitative researcher will explain that we would like to audio-record the interview and explain the processes for ensuring anonymity and confidentiality of interview data.
- The qualitative researcher will explain how interview data will be used.
- The qualitative researcher will determine if the participant would like to take part in the study and if so, will obtain verbal and written consent. If the participant would no longer like to take part they will be thanked for their time and the interview will not proceed.
- Participants will be provided with the opportunity to ask any questions.

This topic guide summarises the main areas to be explored for each interview. As with any qualitative interview, these headings are intended as a starting point to ensure the primary issues are covered, whilst allowing flexibility for new issues to emerge.

Main interview:

- Can you tell me a little bit about the types of patients you see?
    - Types of conditions
    - Stage of condition, treatment, recovery you see them at

    - How common is it for patients for need reconstructive surgery? Is it a last resort?

- What types of surgery or treatment are such patients usually offered?
    - Could you talk me through the process of patient's suffering a condition and being offered treatment? Are there usually different options? Are patients guided in deciding what is best for them? What types of pros/cons do patients usually have to weigh up/decide between?

- What types of difficulties do patients most commonly experience? And do you feel as though this changes throughout treatment and recovery?

- What do you believe are important outcomes for patients?

- What elements of a patient's life do you feel is usually the most negatively affected by the condition, treatment or recovery?

    Areas may include: Socially, family and friends / Interaction with family / Friends
    - Home activities
    - At work
    - Hygiene
    - Hobbies
    - Sleep
    - Intimacy
    - Well-being/how happy they feel
    - Pain & Suffering

- What support do patients usually receive?
    - Acute/rehabilitation?
    - Mental health support?

- Do you believe that a patient's condition, treatment/recovery influences their emotional well-being/how happy they feel? If so in what way and why? How do you think patients cope with this, any strategies they appear to employ or mention to you?
- Do patients mention being concerned about doing anything in the future/any long-term consequences?
- Are patients often over optimistic? Or over pessimistic about recovery?
- Is there anything else you want to tell me?

End of interview

- Thank participant and ask if they have any comments
- Explain again about how data will be used and reiterate about anonymity and confidentiality
- Provide opportunity for questions and state that the lead researcher is contactable after the interview, should questions arise.
