# Supplementary material for: What is important to adults after lower limb reconstruction surgery: a conceptual framework
Source: Qual Life Res. 2023 Jan 7;32(6):1671–82. doi: 10.1007/s11136-022-03340-7 (PMC9825128; doi:10.1007/s11136-022-03340-7)
Supplement: Supplementary file 2 — Supplementary file2 (PDF 214 kb) [file 11136_2022_3340_MOESM2_ESM.pdf]

Supplementary file 2. Summary of each domain and additional quotes.

Summary and additional quotes for the pain domain

**Sub-domains**	**Summary of findings**	**Quotes**
Pain experienced	• All patients experienced pain during their reconstruction and recovery.<br>• Pain impacted patients' ability to undertake activities of daily living ability to work and their emotional well-being.<br>• The level of pain experienced varied between patients, from little pain to still being in pain years after the reconstruction<br>• As patients recovered pain reduced, and mobility and activities of daily living became easier and more enjoyable. Patients also began to feel happier, independent and more confident in their abilities.	"It was too painful and I think I always felt that people were looking at me, so I didn't really like to go out to be looked at". P18<br>"Pain is a big thing. Pain for us and the patient." HCP4
Infection	• Frame patients experienced infections in their pin sites which they found very painful.<br>• Patients worried about infections and the consequences of repeated infections on their recovery.<br>• Infection was also likely to lead short-term reduced mobility.<br>• Patients became familiar with the warning signs of infection and gained confidence in managing the infections.	"Pin site infections are always the big bug bear because people are doing well and then a pin site infection occurs, they're held back, and they think they're doing something wrong." HCP09

Summary and additional quotes for the perception-of-self domain

Sub-domains	Summary of findings	Quotes
Sense-of-self	• Some patients had an altered sense-of-self and felt as though their identity was threatened due to the loss of their daily routine and ability to socialise and undertake their usual hobbies.<br>• Impact on sense of self was often negative but it could have positive implications such as a change in pace of life, appreciating the small things and being more cautious.<br>• Many regained their sense of self as they recovered and some patients made a conscious effort not to be identified by their reconstruction.	"I didn't let it become me. It's a part of me but it isn't me" P32.<br>"Yes, absolutely. It's completely changed my thinking and the way I drive and approach things. Not only about driving, it's life in general. Yeah, it was a life changing event." P06
Appearance	• Many patients felt self-conscious of what the limb looked like due to the frame, scars, size or shape of the leg. It was common for these patients to wear clothes that covered the limb and to avoid crowded places or situations where the limb would be exposed.<br>• Patients often felt fed up of being starred at or asked questions about their limb.<br>• Skin grafts could feel alien to patients and out of place on their limb.<br>• Acceptance of the appearance of the limb after reconstruction was an important step in recovery.	"It does look a bit unsightly with this big lump on the bottom of your let but I don't particularly give a damn what people think in that respect." P12<br>"Some patients have been quite honest and said, "I felt uncomfortable walking around with it on, you know, with people watching and that kind of thing"" HCP08<br>"But they were going to amputate but they chose to do this instead of a leg amputation, so I'm kind of grateful." P32<br>"After having the frame off I still feel quite nervous about going outside. I've not been wearing shorts because I've got scars galore on my leg now and I think the scars caused by the frame, as I say, have set me back a little bit." P18<br>"This piece of meat here that's stuck to my leg, feels like a piece of meat stuck to my leg. It doesn't feel like me ….it's something which is stuck to me." P16

Summary and additional quotes for the work and finances domain

Sub-domains	Summary of findings	Quotes
Ability to work	• Those who did manual labour jobs were more likely to take longer to return to work and some had to find new jobs. Patients became more likely to be able to return to work as they recovered<br>• Those who could work from home or had office based jobs were likely to return to work more quickly. However, some patients said they did not feel in the right mindset to work during this time.<br>• Being unable to work impacted patients emotional well-being (mood and independence), their perception-of-self and could negatively impact their physical functioning if they returned to work too soon.<br>• Returning to work helped patients to reclaim their sense-of-self and independence.<br>• Worries around future career or job prospects increased as the length of recovery time increased.	"I was working at home a little bit and then it's right, you've got to come back now.... They don't seem to understand and like I have to go for 8am, you know, I have to be there when the kids go off on the school bus because I can't drive. I have to be there and it's between 6 and 7 every night when I come home and it's too much, it's too much." P11<br><br>"I find it quite satisfying that line of work looking after people and making a difference to their standard of living. It's made me appreciate more kind of supporting people that have mobility problems just from having experience." P17<br><br>"I just felt that I was in a mind-set where I felt I could [return to work] because obviously at that point we were still online [working from home], and I felt that I could still talk to students and practitioners and things." P26<br><br>"I had to go back into being a chef, so I'm just waiting for a certificate to come through so that I can start working again which will be quite nice." P18
Financial stability	• Most patients were not openly worried about their finances; they stated they were concentrating on getting better. However, worries did begin as their recovery time increased.<br>• Financial stability was afforded to those who had paid sick leave, were retired or had had a compensation/insurance pay out. Emotional well-being was negatively impacted by financial worries.	"I've been very well supported by work regarding, I get paid my full wage and especially because the accident happened at work, I'll get paid my full wage for a minimum of 12 months and I've reassurance basically from the Deputy Chief, he rang me at home to say, if it's 14 months, if it's 16 months, basically when you're back on parade don't worry, you will get paid. Just get better." P22

Summary and additional quotes for the daily lifestyle and functioning domain

Sub-domains	Summary of findings	Quotes
Physical functioning	• The impact on physical functioning differed depending on age, reason for reconstruction and pre-reconstruction mobility. For most their mobility gradually improved as they recovered.<br>• Physical functioning impacted patients pain levels, ability to work and their emotional well-being. It also impacted their ability to wash and dress themselves, to socialise and to sleep.<br>• Overtime most patients regained their mobility through the natural progression of recovery. However, others had not and felt they would not full regain their mobility.	"I think the sort of overriding point that I would probably make would be obviously it's now 18 months along and I'm still not back to how I was before and I'm never going to be 100% but that's one thing I wished I'd have known maybe at the beginning how long it would take to get to the stage." P21<br><br>"Your independence you miss it. Miss to be able to walk and get something." P29
Hygiene and dressing	• Patients often had difficulties washing and dressing themselves. Although, many developed modifications and work arounds to help with this for both washing and dressing which they took a lot of pride in. This also became easier as their physical functioning improved.<br>• Difficulties with hygiene and dressing impacted patients' emotional well-being and their sense of independence.	"But it's quite full on the care really, you know, that I need, you know. I can't do a lot for myself because I can't stand up for very long, you know, I have help to get washed, me and my husband between us do the washing." P15<br><br>"So we unpicked the leg of my tracksuit bottoms and I'd got a shoelace around the bottom of it and then we hit on the idea of a pillow case with a hole in the bottom to put over the cage and then sort of tie my tracksuit bottoms around that. Then I think during the summer I went into one of these stitching shops in the town and got them to sew a triangle in the bottom of my tracksuit bottoms and a pair of jeans. So I've got the most enormous pair of one legged bell bottoms you've ever seen!" P25<br><br>"But everyday simple stuff, having a shower, I worked out I could use a stool that my youngest daughter used to clean her teeth and things on, if I put that into the bath I could get into the bath and if I lowered the shower down I could have a shower, after about 8 weeks it was fantastic." P13

Sleep	• Both quality and quantity of sleep were impacted during the recovery period.<br>• Patients found it harder to get comfortable and to sleep well especially those with an external fixation. Pain medication was a useful aid for getting sleep.<br>• Sleep impacted patients' emotional well-being if they were not well rested.<br>• Patients found that as they recovered sleep became easier as pain reduced. Frame patients got used/adapted to sleeping with the frame on.	"Yeah you just find yourself, surprisingly very tired. Trying to read a book and reading the same page about 6 or 7 times." P22<br><br>"I didn't realise that when I came home that the duvet would hug around the frame and because your leg is suspended inside this metal frame, your leg wants to drop but there's wires holding you up, so your brain starts going…. I'm a restless sleeper and when you roll over I end up with chucks out of my leg from the metal frame. We ended up for a couple of months in separate beds. I didn't want to damage her! We ended up with like two separate duvets, so I just end up taking it all, half a bed with a metal frame round it." P07<br><br>"No, I've always gone to sleep and I put two pillows under me leg and I also had a big duvet where I used to wrap it around which kept it away from the other leg. No it wasn't bad at all." P16
Socialising and hobbies	• Socialising and hobbies were often negatively impacted during the recovery period. This was often due to their physical limitations or just not being in the right mindset for these activities at this time.<br>• The loss of hobbies and social activities negatively impacted patients' emotional well-being. However, most patients were able to return to socialising and their hobbies as recovery progressed.<br>• Engaging in their usual pastimes and hobbies or finding new ones was important to patients and was related to a return to normality, regaining their independence, their sense-of-self and increased emotional well-being.	"I've always been a bit of a geek, so I turned back to kind of a lot more gaming. I have friends that I kind of made from like 2004 and stayed in contact with who do various things and I made sure that I saw people as kind as was possible, went to eat out, cinema, you know, the social aspect is important. If you feel like you become a prisoner in your own home because you're not able to be mobile that's got to be devastating to your mental health." P17<br><br>"I'm currently about to start in September a university course. I've been studying, reading a lot more … I think becoming more literate than more active sort of thing which has been quite nice." P18<br><br>"It restricts you greatly to be truthful on your social life because you visually become a point to look at don't you and I think sometimes, a few times, if I had to explain one more time, you know. I ended up making jokes about it, you know, if I stood still long enough someone

		would chain a bike to me or if I put my foot up in the air, I'd get free wi-fi. You don't want to go out because you don't want to face the same conversation over again." P07

Summary and additional quotes for the emotional well-being domain

**Sub-domains**	**Summary of findings**	**Quotes**
Mood	• Low mood was experienced by many at some point during recovery, this tended to be feeling depressed, unhappy, sad, isolated and frustrated or guilty at placing a burden on relatives.<br>• Low mood was often influenced by pain levels, frustrations around their physical limitations and feeling unsupported and unknowledgeable about their recovery pathway.<br>• For most patients most mood issues resolved as they recovered.	"Just the whole thing was just….I just felt a whole year out of my life was totally, well I don't know what the word is really, it was just horrendous." P11<br><br>"I want to get back to normality, you know, waking up, going to work, looking forward to having the weekend off and just get back to normal life, you know, instead of just having nothing. Nothing to get up for." P19<br><br>"Just being on hold for two years. Like seeing everybody else buy houses and, I don't know, and you kind of get…. not left behind, I know everybody has wasted a year because of COVID but I still got left behind in a way. Like everybody else is, I don't know, just in front of me kind of thing." P19
Self-efficacy and independence	• Anxiety was common regarding recovery, re-injury or any long term consequences.<br>• Patients often felt very dependent on others, especially during the early stages of their recovery. They also often felt as though their lives were on hold and they had lost or wasted time.<br>• Being resilient and setting goals were seen as important ways to stay positive and optimistic about recovery.<br>Reduced self-efficacy and independence negatively impacted patients daily lifestyle and functioning and their perception-of-self.	"Yeah. I am a strong person and, you know, if there's a problem….if there's a physical problem I will try and find a way around it". P08<br><br>"I think a lot of them say physio helps because they've got something to focus on and we set long and short term goals. So I think if you've got a short term goals then they stay focused and they manage to cope with it because they can see that they're progressing." HCP18

	• Feelings of confidence increased as patients recovered and regained greater mobility. This led to increased independence and reduced anxieties.	"It just feels absolutely great….. there are things now that I can do that I couldn't do sort of like walk further, drive and all of those things that sort of aid your independence."P26<br><br>"I don't know how I did it actually but you just do it don't you because you've got no option." P10<br><br>"Oh well I mean I think that [re-injury] will be a huge anxiety to be honest. Well I think I am worried about falling, you know, because obviously having two operations in 6 months is a lot to get round isn't it about falling. So, you know, I mean it was just a silly accident that could happen to anybody both times, but I will have to personally be more careful because I don't want to go through this again." P24<br><br>"I was the doer and all of a sudden, I became the do'ee and my brain didn't like that…it does your head in, in the fact that you've got to sit there and be an observer rather than a doer." P07

Summary and additional quotes for the support domain

**Sub-domains**	**Summary of findings**	**Quotes**
Support from health professionals, friends and family and work	• Feeling supported both acutely and longer term by health professionals was very important to patients.<br>• Supported patients often felt more relaxed about their recovery pathway. It was also important for patients to feel informed about their reconstruction and what to expect during recovery.<br>• Those with a close network of family members and friends felt better supported during their recovery.<br>• Strong support systems could cause the patient to feeling guilty at the increased burden they were placing on others.<br>• Support from work reduced patients' worries surrounding their future employment and financial problems.	"One of my experiences is that a nut came off my frame maybe 2 or 3 weeks after I came home and I didn't have a clue what to do. I didn't know who to get in touch with. So it was just by trial and error that, you know, I was ringing the hospital, I was ringing trauma out-patients, ringing the physios, ringing the hospital ward which is very difficult by the way to get an answer from anybody. So it took me actually 2 days to get in touch with anybody regarding that and I needed to go back into hospital to have it put back on." P24<br>"I'm still waiting two weeks on to hear and speak to Dr [name] and I can understand that he may be busy, but I just feel like I've been left behind." P18

	• Support eased patients' recovery and reduced worries around the impact on work and finances, and daily lifestyle and functioning. It also positively impacted patient's emotional well-being as it enabled them to focus on their recovery.	"If they can get all of those things in place [support], they do tend to improve if you like but definitely those that have those challenges compared to those that haven't, you know, have a more supportive home life, you know, have friends and family close by and are more financially secure if you like. They seem to have a much better patient care than those that don't." HCP19<br><br>"My wife's free time, has dramatically decreased to almost zero. She has me to look after and I'm basically taking a lot of her time and it's a lot of effort….I was fully dependent on her."P06<br><br>"They're waiting for me [employers]. They've even sent me a card signed by everyone and so on. It's really nice of them. They miss me." P06
